# *Burkholderia pseudomallei* biofilm resists *Acanthamoeba* sp. grazing and produces 8-*O*-4′-diferulic acid, a superoxide scavenging metabolite after passage through the amoeba

**DOI:** 10.1038/s41598-023-43824-1

**Published:** 2023-10-03

**Authors:** Chainarong Bunma, Parumon Noinarin, Jutarop Phetcharaburanin, Sorujsiri Chareonsudjai

**Affiliations:** 1 Department of Microbiology, Faculty of Medicine, Khon Kaen University, Khon Kaen, Thailand; 2 Department of Occupational Health and Safety, Faculty of Public Health, Nakhon Ratchasima Rajabhat University, Nakhon Ratchasima, Thailand; 3 Department of Systems Biosciences and Computational Medicine, Faculty of Medicine, Khon Kaen University, Khon Kaen, Thailand; 4 Khon Kaen University Phenome Center, Faculty of Medicine, Khon Kaen University, Khon Kaen, Thailand; 5 Cholangiocarcinoma Research Institute, Khon Kaen University, Khon Kaen, Thailand; 6 Research and Diagnostic Center for Emerging Infectious Diseases (RCEID), Khon Kaen, Thailand

**Keywords:** Bacteria, Biofilms

## Abstract

*Burkholderia pseudomallei*, an etiological agent of melioidosis is an environmental bacterium that can survive as an intracellular pathogen. The biofilm produced by *B. pseudomallei* is crucial for cellular pathogenesis of melioidosis. The purpose of this investigation is to explore the role of biofilm in survival of *B. pseudomallei* during encounters with *Acanthamoeba* sp. using *B. pseudomallei* H777 (a biofilm wild type), M10 (a biofilm defect mutant) and C17 (a biofilm-complemented strain). The results demonstrated similar adhesion to amoebae by both the biofilm wild type and biofilm mutant strains. There was higher initial internalisation, but the difference diminished after longer encounter with the amoeba. Interestingly, confocal laser scanning microscopy demonstrated that pre-formed biofilm of *B. pseudomallei* H777 and C17 were markedly more persistent in the face of *Acanthamoeba* sp. grazing than that of M10. Metabolomic analysis revealed a significant increased level of 8-*O*-4′-diferulic acid, a superoxide scavenger metabolite, in *B. pseudomallei* H777 serially passaged in *Acanthamoeba* sp. The interaction between *B. pseudomallei* with a free-living amoeba may indicate the evolutionary pathway that enables the bacterium to withstand superoxide radicals in intracellular environments*.* This study supports the hypothesis that *B. pseudomallei* biofilm persists under grazing by amoebae and suggests a strategy of metabolite production that turns this bacterium from saprophyte to intracellular pathogen.

## Introduction

*Burkholderia pseudomallei* is an etiological agent of melioidosis. This bacterium is generally an environmental saprophyte dwelling in soil and water^1–5^. This pathogen can be transmitted to susceptible human hosts via ingestion, inhalation, or skin inoculation. It can become an intracellular pathogen, evading host immune surveillance using numerous virulence strategies and contribute to its pathogenicity and disease severity, resulting in mortality rates that range from 40 to 70%^6–9^. Melioidosis is of growing public health concern, causing an estimated 165,000 cases and 89,000 deaths per year^10^. Recently, there has been a call for WHO to officially recognise melioidosis as a neglected tropical disease^11^. There is clearly a need to understand how this saprophytic bacterium evolved to become a life-threatening pathogen.

*Burkholderia pseudomallei* can persist in non-living reservoirs, including distilled water, for 16 years^12^ and remains viable in a soil microcosm for at least 120 days^2^. The bacterium can also live in other living organisms including grasses^13^ and free-living amoebae from the genus *Acanthamoeba*^14^. Biofilm formation is a key factor for bacterial survival in diverse natural environments and in interactions with the host^15^. Biofilm formation by *Pseudomonas aeruginosa* and *Vibrio cholerae* facilitates their survival and persistence in the environment despite grazing by protozoans^16,17^. Previous research has established that *B. pseudomallei* biofilm promotes bacterial adhesion and internalisation in human epithelial A549 cells^18^. It has not yet been determined whether *B. pseudomallei* biofilm plays a role in interactions with living organisms other than human hosts.

*Acanthamoeba* offers a model for the development of intracellular pathogenicity in humans as they facilitate the intracellular survival of pathogens within themselves^19,20^. Both human professional phagocytes and amoebae produce reactive oxygen and nitrogen species such as nitric oxide (NO), superoxide (O_2_^–^) and hydrogen peroxide (H_2_O_2_) as antimicrobial molecules^21,22^. It has previously been observed that hypervirulent *V. cholerae* evolution after passing through protozoan predation acquired strategies to reduce intracellular stress responses including superoxide (O_2_^−^) and H_2_O_2_^23,24^. Moreover, previous research has established that superoxide dismutase C production is essential to provide resistance against killing by reactive oxygen intermediates, leading to intracellular survival in host phagocytes and hence virulence of *B. pseudomallei*^12^. Much uncertainty remains about the intracellular adaptations that allow environmental *B. pseudomallei* to become an intracellular pathogen after interacting with organisms in the environment, including *Acanthamoeba* spp*.*

**Figure 1:**
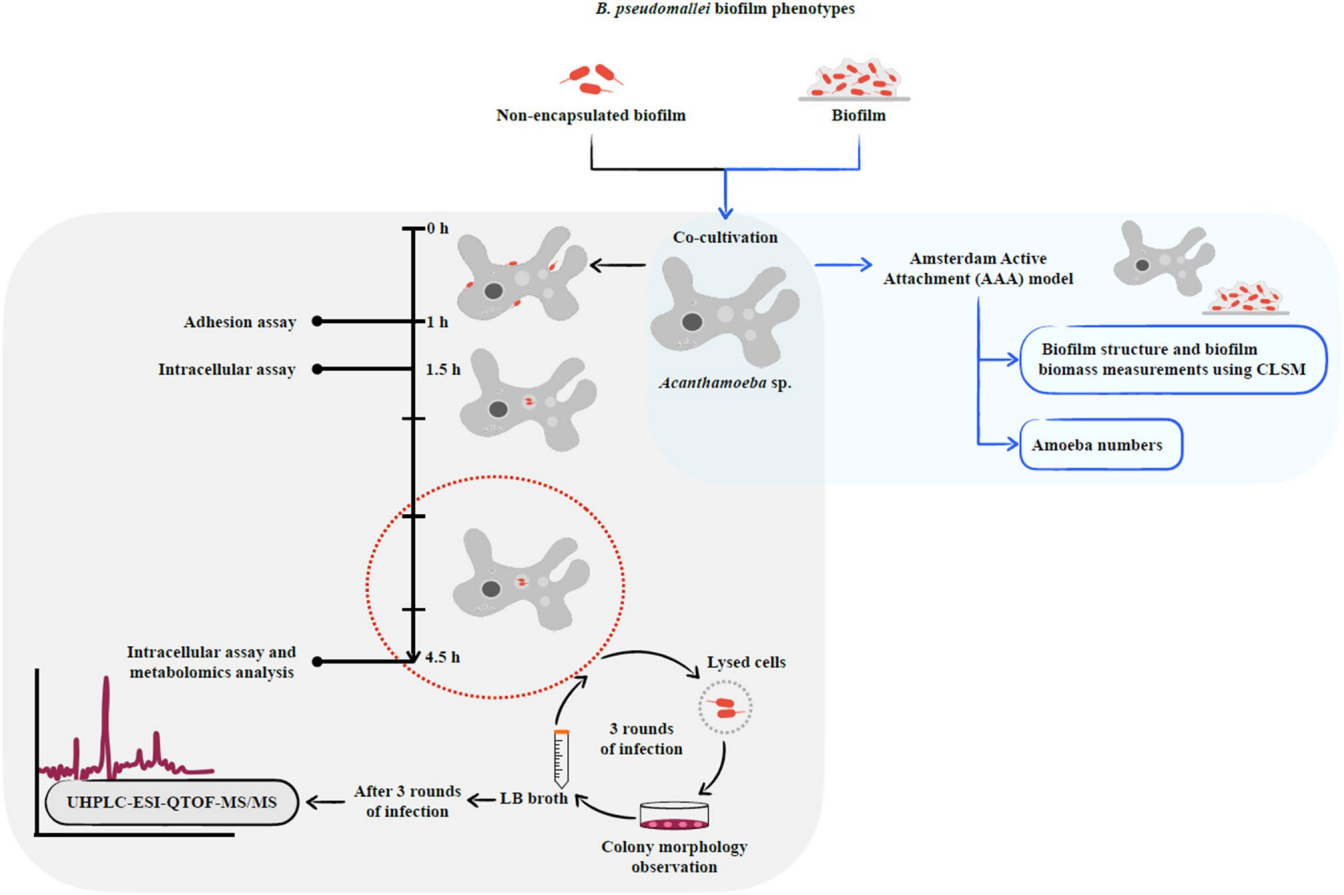
Schematic flow chart of the co-cultivation of two different *B. pseudomallei* biofilm phenotypes and *Acanthamoeba* sp. Adhesion and intracellular-survival assays at MOI 100 using non-encapsulated biofilm cells were performed. *Burkholderia pseudomallei* were passaged through *Acanthamoeba* sp. up to three times and were then collected for metabolomic analysis using ultra-high-performance liquid chromatography-electrospray ionization-quadruple time-of-flight mass spectrometry in parallel with observations of colony morphology on Ashdown’s agar. In addition, preformed 24-h and 48-h *B. pseudomallei* biofilm was cocultured with *Acanthamoeba* sp. to monitor the biofilm structure and biofilm biomass using confocal laser scanning microscopy. Amoeba cells were counted using a hemocytometer.

We investigated this question by co-cultivation of *B. pseudomallei* H777 (a clinical isolate, moderate biofilm producing wild-type), M10 (a biofilm-defect mutant of H777) and C17 (a biofilm-complemented of M10) (Table 1) with *Acanthamoeba* sp. to clarify the role of biofilm on *B. pseudomallei-*amoeba interaction. Bacterial adhesion, intracellular survival, metabolomic analyses of serially passaged *B. pseudomallei*, and persistence of bacterial biofilm grazed by amoebae were all investigated (Fig. 1). Our results demonstrated the persistence of *B. pseudomallei* biofilm formation against grazing by amoebae. The importance and originality of this study is that it explores for the first time the intracellular superoxide scavenger metabolites produced by *B. pseudomallei* following encounters with amoebae and demonstrates the persistence of *B. pseudomallei* biofilm despite grazing by amoebae. The presence of superoxide scavenger metabolites following passage through amoebae may indicate a pathway by which *B. pseudomallei* can become hypervirulent and a human pathogen.

**Table 1:** *Burkholderia pseudomallei* strains.

Strains	Characteristics	Sources/description	Antibiotic supplemented	References
H777	Moderate biofilm formation	Blood of melioidosis patient, Srinagarind Hospital, Khon Kaen, Thailand	None	^49^
M10	Biofilm-defective mutant of H777	Tn5-OT182 mutant to inactivate *bpsl0618*, a sugar transferase gene	Tetracycline 50 µg/mL	^49^
C17	Biofilm-complemented of M10	Function of *bpsl0618* restored	Tetracycline 50 µg/mL Chloramphenicol 30 μg/mL	^18^

## Results

### Non-encapsulated biofilm cells of *B. pseudomallei* H777 and M10 showed similar adhesion to and survive within *Acanthamoeba* sp. cells

**Figure 2:**
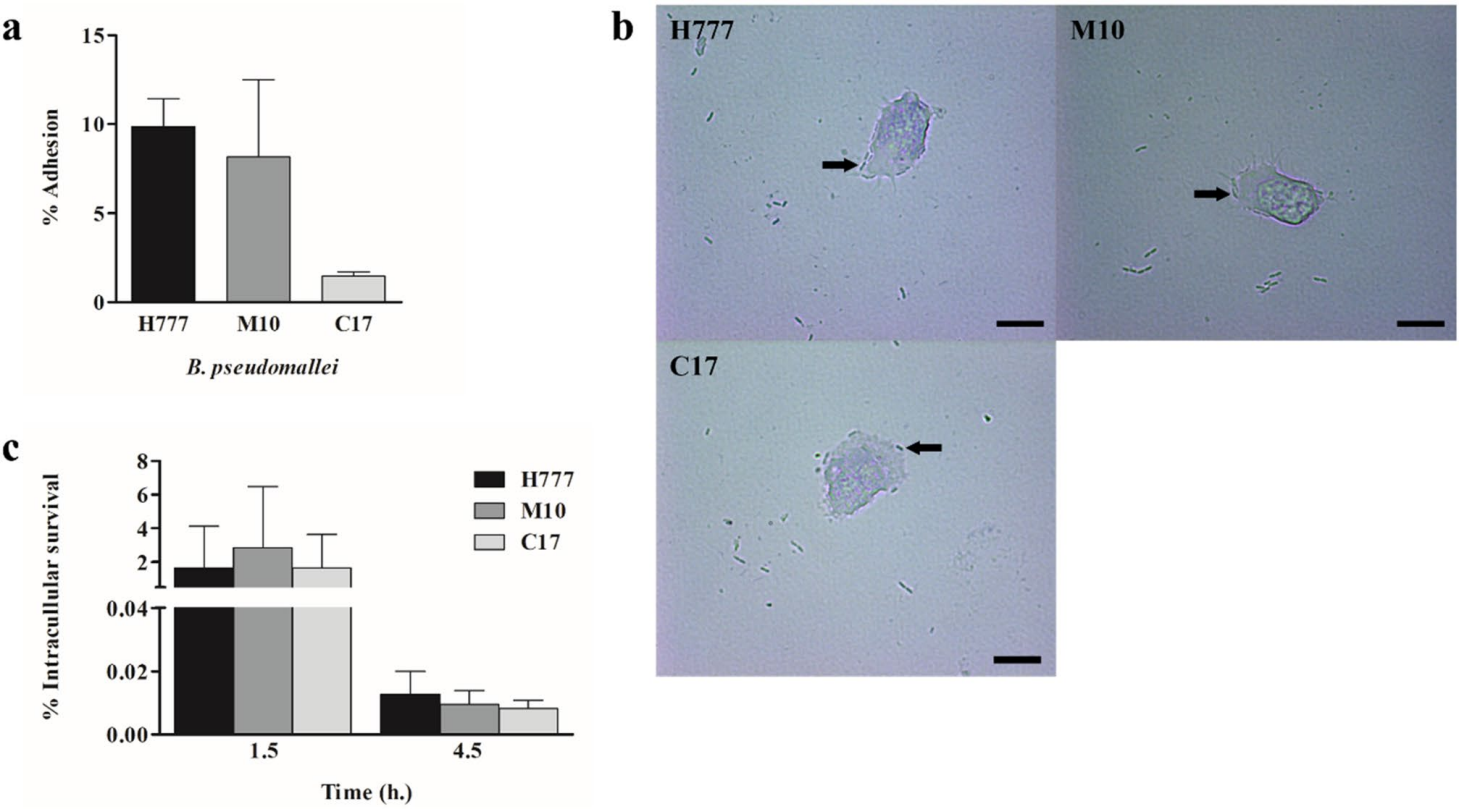
Percentage of *B. pseudomallei* cells adhering to amoebae and intracellular survival after co-cultivation within *Acanthamoeba* sp. Similar percentages of planktonic *B. pseudomallei* H777 and M10 cells adhered to *Acanthamoeba* sp. after 1 h but not in the case of C17 (**a**). Bright field microscope visualization of *B. pseudomallei* H777, M10 and C17 adhering to *Acanthamoeba* sp. (black arrow) at 1,000 × magnification, scale bars = 10 µm (**b**); Cells of *B. pseudomallei* H777, M10 and C17 exhibited similar internalised at the early phase of infection at 1.5 h post-infection (p.i.) in *Acanthamoeba* sp. and similar survived at 4.5 p.i. (**c**). The experiment was performed in four replicates in three independent experiments (n = 12). Error bars represent mean ± SD. Statistical significance was tested using one-way ANOVA followed by Tukey post hoc test.

Non-encapsulated biofilm cells of *B. pseudomallei* H777 and M10 cells co-cultured with *Acanthamoeba* sp. at MOI 100 for 1 h revealed similar levels of bacterial adhesion to *Acanthamoeba* sp.. However, this was not the case for the C17 strain (Fig. 2a and b). After the kanamycin protection assay, the number of *B. pseudomallei* of all three strains within the amoebae exhibited comparable levels at 1.5 h p.i. and 4.5 h p.i. (Fig. 2c).

Non-encapsulated biofilm cells of *Burkholderia pseudomallei* H777, M10 and C17 co-cultured with *Acanthamoeba* sp. at MOI 100 for 1 h were then monitored under an inverted microscope for another 10 min. The results revealed the amoeba exhibited grazing actions on bacterial cells (Supplement video 1).

### *Burkholderia pseudomallei* biofilm persist to *Acanthamoeba* sp. predation

**Figure 3:**
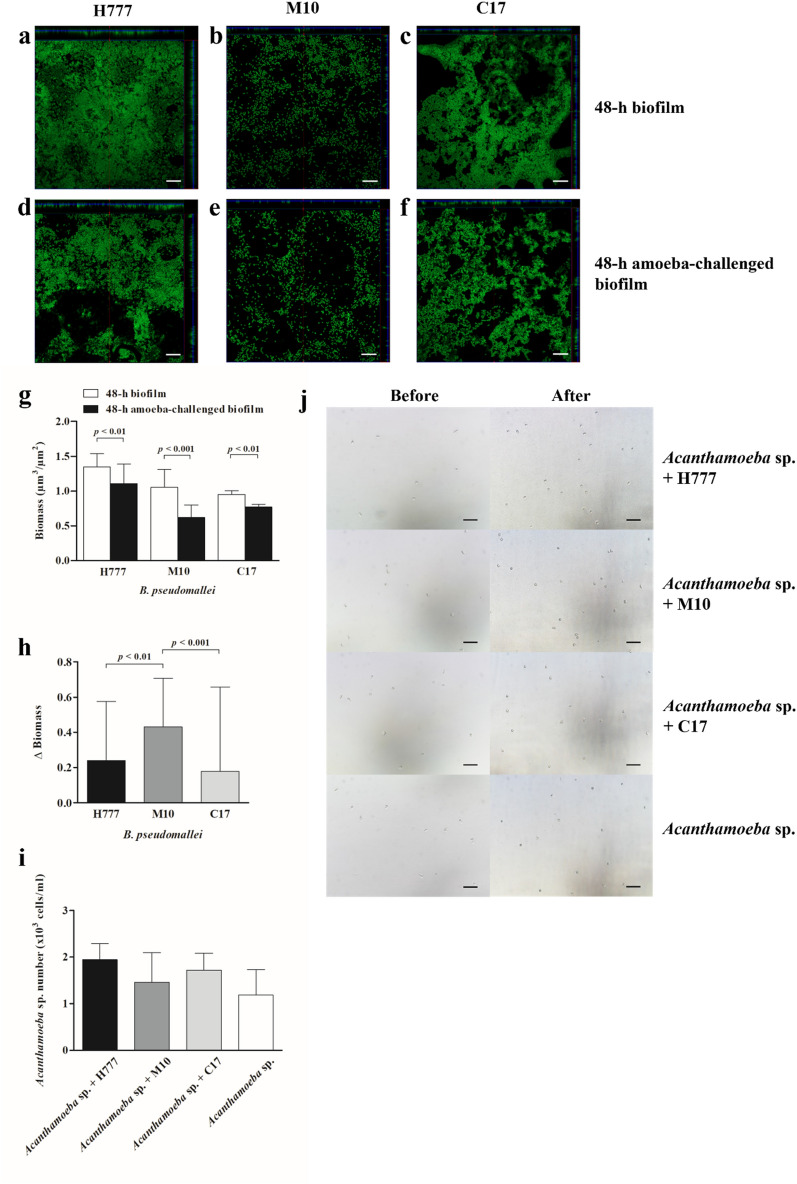
The 48-h *B. pseudomallei* H777, M10 and C17 biofilms after challenged with *Acanthamoeba* sp. The 24-h *B. pseudomallei* H777, M10 and C17 pre-formed biofilms were co-cultured with *Acanthamoeba* sp. for 24 h and then biofilm structure and biomass were assessed by CLSM, and numbers of amoebae counted using a hemocytometer. CLSM images of the 48-h *B. pseudomallei* H777, M10 and C17 biofilms (**a**–**c**). CLSM images of the 48-h amoeba-challenged *B. pseudomallei* H777, M10 and C17 biofilms (630 × magnification, scale bars = 10 µm.) (**d**–**f**). Biomass was compared between the co-cultured biofilms with amoebae and controls (**g**). Δ Biomass of *B. pseudomallei* H777, M10 and C17 after co-cultivation with amoebae (**h**). The biomass and Δ Biomass data from 72 images (24 image z-stacks from 4 cover slips in three independent experiments) were used in each analysis. Numbers of amoeba cells after incubation with bacterial biofilm from duplicates of the three independent experiments (n = 6) (**i**). Amoeba population after co-cultivation the biofilms (100 × magnification, scale bars = 50 µm) (**j**). Error bars represent mean ± SD. Statistical significance was tested using one-way ANOVA followed by Tukey post hoc test.

**Figure 4:**
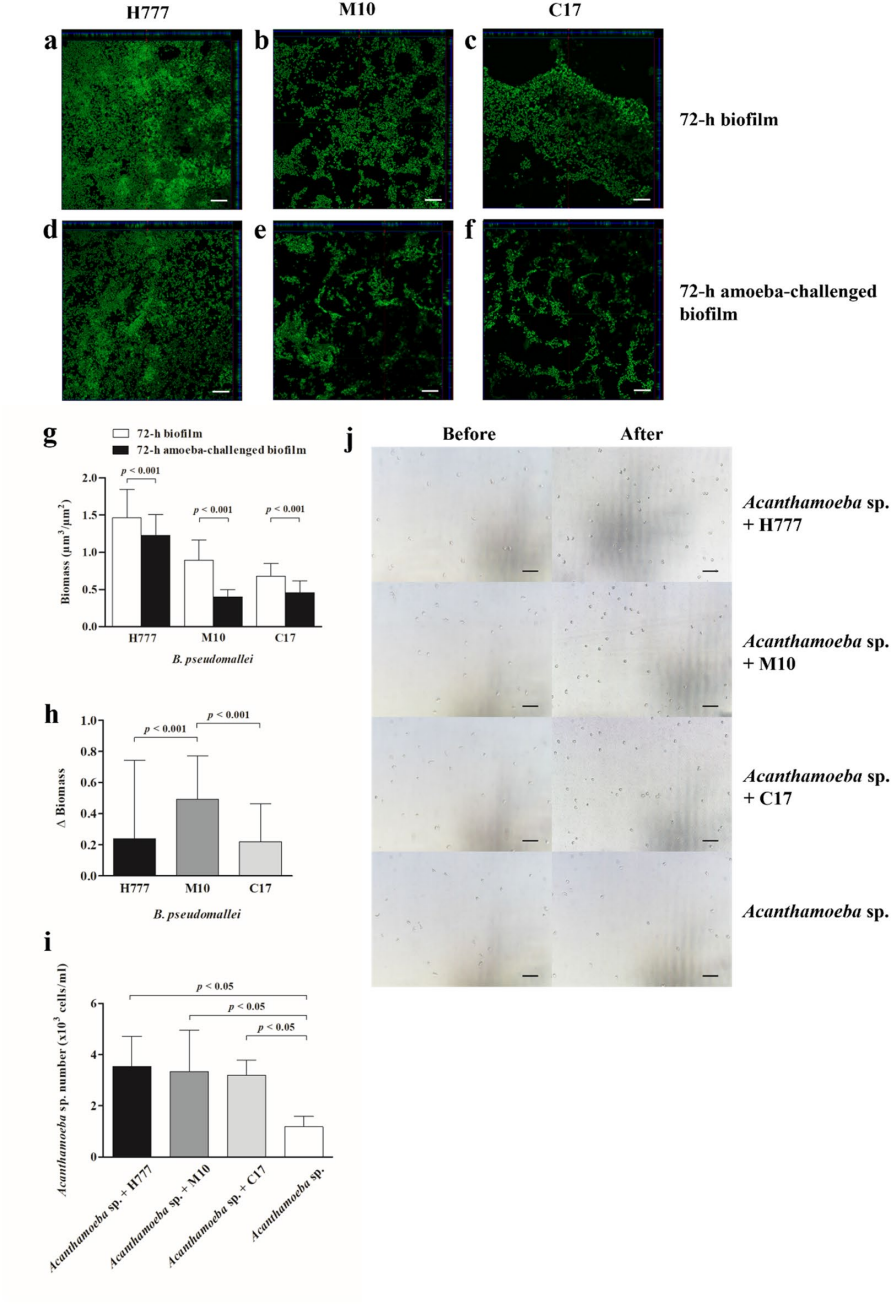
The 72-h *B. pseudomallei* H777, M10 and C17 biofilms after challenged with *Acanthamoeba* sp. The 48-h *B. pseudomallei* H777, M10 and C17 biofilms were co-cultured with *Acanthamoeba* sp. for 24 h and then biofilm structure and biomass were assessed by CLSM, and numbers of amoebae counted using a hemocytometer. CLSM images of the 72-h *B. pseudomallei* H777, M10 and C17 biofilms (**a**-**c**). CLSM images of the 72-h amoeba-challenged *B. pseudomallei* H777, M10 and C17 biofilms (630 × magnification, scale bars = 10 µm.) (**d**–**f**). Biomass was compared between the co-cultured biofilms with amoebae and controls (**g**). Δ Biomass of H777, M10 and C17 after co-cultivation with amoebae (**h**). The biomass and Δ Biomass data from 72 images (24 image z-stacks from 4 cover slips in three independent experiments) were used in each analysis. Numbers of amoeba cells after incubation with bacterial biofilm from duplicates of the three independent experiments (n = 6) (**i**). Amoeba population after co-cultivation the biofilms (100 × magnification, scale bars = 50 µm) (**j**). Error bars represent mean ± SD. Statistical significance was tested using one-way ANOVA followed by Tukey post hoc test.

To investigate the persistence of *B. pseudomallei* biofilm despite grazing by amoebae, the 24-h and 48-h preformed biofilms of *B. pseudomallei* H777, M10 and C17 were co-cultured with *Acanthamoeba* sp. for an additional 24 h. Confocal images and COMSTAT analysis revealed the disruption of the 48-h amoeba-challenged biofilm biomass of both *B. pseudomallei* H777, M10 and C17 (Fig. 3a–g) and the 72-h amoeba-challenged biofilm biomass of both *B. pseudomallei* H777, M10 and C17 (Fig. 4a–g) compared to the untreated control (*p* < 0.01, 0.001). Notably, *B. pseudomallei* H777 and C17 biofilms were better able persist against grazing by amoebae than was that of M10 (Figs. 3h and 4h). The numbers of amoebae when co-cultured with the 48-h *B. pseudomallei* H777, M10 and C17 biofilms, amoebae numbers were comparable to those cultured alone (Fig. 3i,j). However, the co-cultured with the 72-h *B. pseudomallei* H777, M10 and C17 biofilms resulted in a significant increase in amoebae numbers to amoebae cultured alone (*p* < 0.05) (Fig. 4i,j). These findings suggest that the 72-h *B. pseudomallei* biofilms may serve as a food source for *Acanthamoeba* sp., leading to the increased amoeba cell numbers.

### Metabolic phenotypes of internalised *B. pseudomallei* H777 after three passages through *Acanthamoeba* sp

To monitor the metabolic alterations in the repeatedly internalised *B. pseudomallei* in *Acanthamoeba* sp., *B. pseudomallei* H777 was grown on Ashdown's agar after three passages through amoebae. The colony morphology of *B. pseudomallei* H777 liberated from each passage was similar to the control (Supplement Fig. 1a, b).

**Figure 5:**
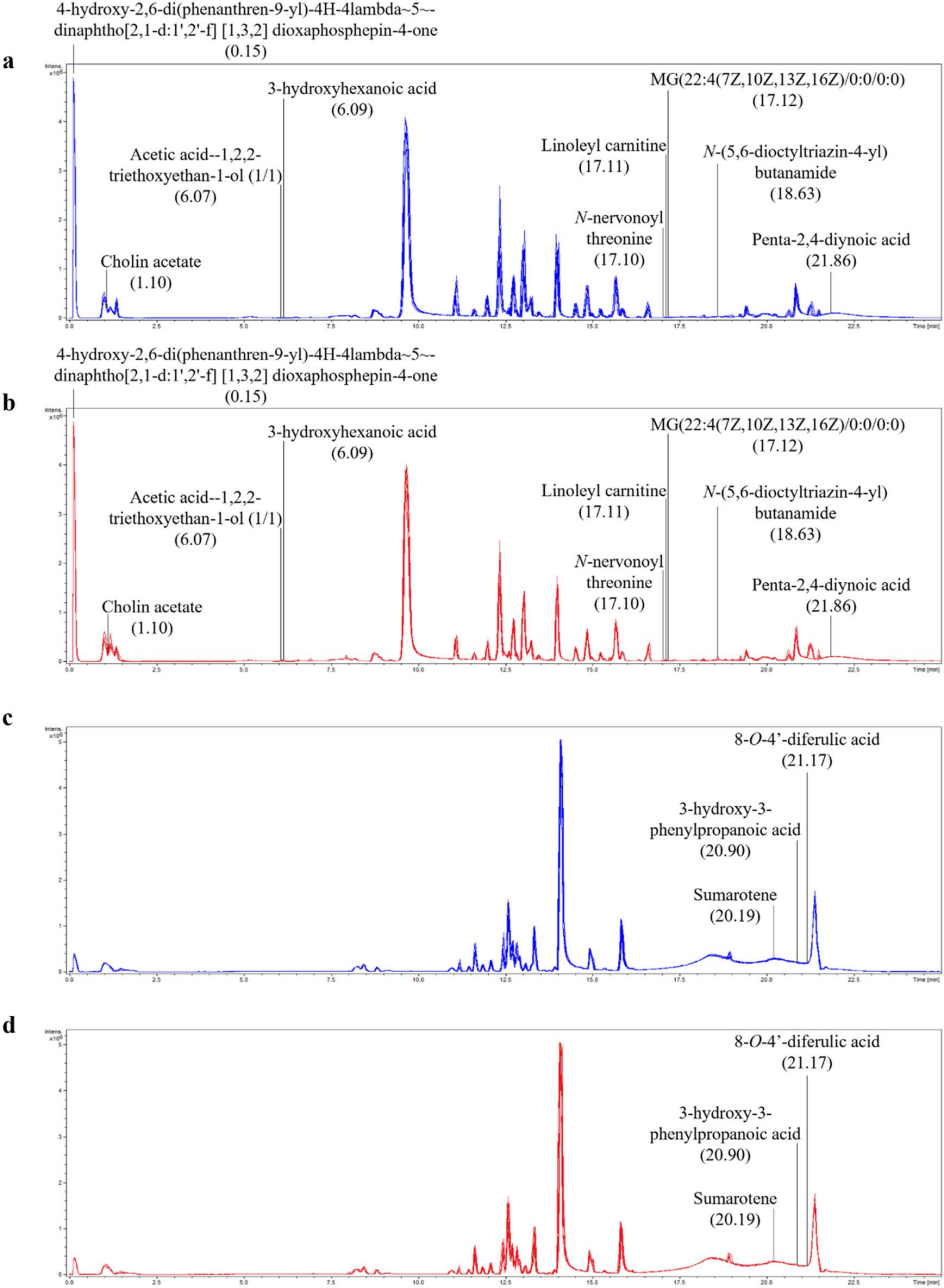
Untargeted profile chromatogram of MS/MS spectra intensities with retention time. Untargeted profile chromatogram of MS/MS spectra intensities with retention time in positive (**a** and **b**) and negative (**c** and **d**) ionisation mode. Blue peak, *B. pseudomallei* H777 without *Acanthamoeba* sp. (n = 5). Red peak, *B. pseudomallei* H777 co-cultured with *Acanthamoeba* sp. (n = 5).

The intracellular metabolites following three passages of *B. pseudomallei* in *Acanthamoeba* sp. were subjected to UHPLC-ESI-QTOF-MS/MS analysis. Peak chromatography with retention time in positive and negative ESI modes showed no differences between experimental and control groups (Fig. 5).

The metabolome datasets contained 1587 and 1052 features in positive and negative ESI modes, respectively (data not shown). Subsequently, PCA and O-PLS-DA models were constructed using Pareto the scaling method. PCA scores plot revealed that the metabolic profiles of *B. pseudomallei* and control groups were generally similar in both positive and negative ESI modes (Supplement Fig. 2a,b). In addition, the O-PLS-DA models showed no significant difference between groups in positive (*p* = 0.43) and negative (*p* = 0.83) ESI modes (Supplement Figs. 2c,d).

Relative concentrations of identified metabolites in each ESI mode analysed using fold-change with cut-off > 1.2 (Table 2). Nine metabolites of positive ESI mode including 3-hydroxyhexanoic acid (C_6_H_12_O_3_), *N*-(5,6-dioctyltriazin-4-yl) butanamide (C_23_H_42_N_4_O), 4-hydroxy-2,6-di (phenanthrene-9-yl)-4H-4lambda ~ 5 ~ -dinaphtho[2,1-d:1′,2′-f][1,3,2]dioxaphos -phepin-4-one (C_48_H_29_O_4_P), *N*-nervonoyl threonine (C_28_H_53_NO_4_), penta-2,4-diynoic acid (C_5_H_2_O_2_), MG(22:4(7Z,10Z,13Z,16Z)/0:0/0:0) (C_25_H_42_O_4_), cholin acetate (C_7_H_17_NO_3_), linoleyl carnitine (C_25_H_45_NO_4_), and acetic acid—1,2,2-triethoxyethan-1-ol (1/1) (C_10_H_22_O_6_) were significantly observed in internalised *B. pseudomallei* compared to that in LB growth. In addition, three metabolites of negative ESI mode including 8-*O*-4′-diferulic acid (C_14_H_10_Cl_4_), sumarotene (C_24_H_30_O_2_S), and 3-hydroxy-3-phenylpropanoic acid (C_9_H_10_O_3_) were detected.

**Table 2:** Metabolites exhibiting > 1.2-fold change between *B. pseudomallei* before and after the passage through the amoeba.

RT (min)	m/z	Metabolite name	Formula	Adduct	Exact mass	LoA*	Mean Cont	Mean Bp	Fold change	*p*-value**
6.09	133.08701	3-hydroxyhexanoic acid	C_6_H_12_O_3_	[M + H] +	132.0786	1	279.8	1901.8	5.80	0.16
18.63	391.34161	*N*-(5,6-dioctyltriazin-4-yl) butanamide	C_23_H_42_N_4_O	[M + H] +	390.3359	1	8208.4	36,533.4	3.45	0.42
8.01	171.10171	Unknown 1	–	–	–	–	249	1077.4	3.33	–
0.21	758.87048	Unknown 2	C_30_H_14_O_10_S_7_	[M + H] +	757.8632	1	434.2	1817.6	3.19	–
18.63	452.39433	Unknown 3	C_20_H_49_N_7_O_4_	[M + H] +	451.3846	1	6320.2	26,236.2	3.15	–
18.62	496.42111	Unknown 4	–	–	–	1	9754.2	28,398.4	1.91	–
0.15	701.18885	4-hydroxy-2,6-di(phenanthren-9-yl)-4H-4lambda ~ 5 ~ -dinaphtho[2,1-d:1',2'-f] [1, 2, 3] dioxaphosphepin-4-one	C_48_H_29_O_4_P	[M + H] +	700.1803	1	656.4	1699	1.59	0.06
17.10	468.39039	*N*-nervonoyl threonine	C_28_H_53_NO_4_	[M + H] +	467.3975	1	6194.6	15,083.8	1.43	0.42
21.86	95.01233	Penta-2,4-diynoic acid	C_5_H_2_O_2_	[M + H] +	94.0055	1	496.2	1206.6	1.43	0.14
18.52	80.94756	Unknown 5	–	–	–	1	1334.8	3199.8	1.40	–
13.24	407.143	Unknown 6	–	–	–	1	787.9	1864.2	1.37	–
17.12	407.33609	MG(22:4(7Z,10Z,13Z,16Z)/0:0/0:0)	C_25_H_42_O_4_	[M + H] +	406.3083	1	4697.8	11,079.8	1.36	0.31
1.10	164.12822	Cholin acetate	C_7_H_17_NO_3_	[M + H] +	163.1208	1	24,875.4	58,152.4	1.34	0.31
17.11	424.36421	Linoleyl carnitine	C_25_H_45_NO_4_	[M + H] +	423.3349	1	6279	14,568.8	1.32	0.22
6.07	239.14915	Acetic acid–1,2,2-triethoxyethan-1-ol (1/1)	C_10_H_22_O_6_	[M + H] +	238.1416	1	9161.8	20,853.2	1.28	0.02
0.19	622.8926	Unknown 7	–	–	–	–	341.2	1644	3.82	–
21.17	316.94843	8-*O*-4'-diferulic acid	C_14_H_10_Cl_4_	[M–H]-	317.9537	1	4417	18,930.2	3.29	0.04
19.54	89.02384	Unknown 8	–	–	–	–	3431.2	13,719.6	3.00	–
21.18	520.9089	Unknown 9	C_11_H_5_N_6_O_13_P_3_	[M–H]-	521.9128	1	2457.8	9463.4	2.85	–
20.19	381.17699	Sumarotene	C_24_H_30_O_2_S	[M–H]-	382.1967	1	508.4	1518.6	1.99	0.01
0.18	698.86868	Unknown 10	–	–	–	–	671.8	1652.4	1.46	–
20.90	165.01959	3-hydroxy-3-phenylpropanoic acid	C_9_H_10_O_3_	[M–H]-	166.0630	1	609	1408.6	1.31	0.11

*Levels of Assignment (LoA) including (1) accurate mass matched to database, (2) accurate mass matched to database and tandem MS spectrum matched to in silico fragmentation pattern, (3) tandem MS spectrum matched to database or literature, (4) retention time and the molecular mass matched to standard compound, and (5) MS/MS spectrum matched standard compound.

**Mann–Whitney U test (n = 5 in each group, *p* < 0.05).

Bp:  *Acanthamoeba* sp.- internalized *Burkholderia pseudomallei*, Cont.: *Burkholderia pseudomallei* in LB broth.

In addition, we also performed univariate analysis to compare spectral intensities between *B. pseudomallei* and the control group of 12 metabolites using the Mann–Whitney U test (Table 2). The results revealed that acetic acid-1,2,2-triethoxyethan-1-ol (1/1) (*p* = 0.02), 8-*O*-4′-diferulic acid (*p* = 0.04), and sumarotene (*p* = 0.01) in *B. pseudomallei* were significantly different compared to the control group.

## Discussion

*Burkholderia pseudomallei* is an environmental bacterium that thrives in soil and water, commonly establishes interactions with a variety of organisms, including plants and amoebae, particularly in melioidosis-endemic regions. Bacterial biofilm plays a crucial role in the survival of bacteria in diverse environments and contributes to their ability to cause diseases in human hosts. This study set out with the aim of assessing the role of *B. pseudomallei* biofilm on its survival against grazing by amoebae. The metabolic differences between the amoeba-internalised *B. pseudomallei* and control cultures were analysed. The results revealed that bacterial biofilm was dispersed after co-cultivation, but the *B. pseudomallei* biofilm-forming strain H777 and C17 persisted better against *Acanthamoeba* sp. grazing than did the biofilm-defect mutant strain, M10. A possible explanation for this finding might be that biofilm formation partly protects against grazing by amoebae. Furthermore, a significant increased level of 8-*O*-4′-diferulic acid, a superoxide scavenging metabolite, from the *B. pseudomallei* cells passaged three times in *Acanthamoeba* was observed*.* Hence, it could conceivably be hypothesized that grazing pressure from free-living amoebae may serve as a “training ground” stimulating the environmental saprophytic *B. pseudomallei* to produce compounds and may assist its survival in host cells.

Environmental saprophytes are commonly constrained by protozoan predation in natural food webs^16^. Biofilm formation provides bacterial cells with some shelter from these threats^15^. The persistence of the *B. pseudomallei* wild-type biofilm against grazing by *Acanthamoeba* sp. broadly supports the role of biofilm as an antipredator mechanism. The opportunistic bacterial pathogen, *Vibrio cholerae*, can survive protozoan grazing in biofilm form while non-encapsulated biofilm cells are eliminated. The environmental persistence of *V. cholerae* biofilms correlated with the principal cause of seasonal cholera epidemics^16^. Furthermore, the *P. aeruginosa* biofilms were demonstrated effectively defended against *A. castellanii* grazing^25^. This opportunistic pathogenic, *P. aeruginosa* was exhibited the type 3 secretory system components to kill biofilm-associated amoebae and may associate with the evolution of opportunistic bacterial pathogens^26^.

In this study, different biofilm phenotypes of *B. pseudomallei* (H777, M10 and C17) were apparently grazed and used as food by *Acanthamoeba* sp. as indicated by increased numbers of amoebae. This finding is consistent with our previous results on predator–prey relationships between *B. pseudomallei* and *Acanthamoeba* sp.^27^ and broadly supports the work of other studies in this area linking bacteria and *Acanthamoeba* sp.. *Acanthamoeba castellanii* was demonstrated as a biofilm grazer of mixed biofilms communities of *Klebsiella pneumoniae*, *P. fluorescens* and *S. epidermidis*^28^. Cell-free supernatant of *A. castellanii, A. lenticulate* and *A. polyphaga* disrupted the preformed biofilms of methicillin-resistant *Staphylococcus aureus* and *Mycobacterium bovis*. Biofilm dispersion by predatory amoebae highlights the potential for biofilm-busting, suggesting the possibility of identifying active molecules that can be applied as novel anti-biofilm compounds for management of biofilm-associated infections in conjunction with antimicrobial agents^29^. A possible explanation for the higher number of *Acanthamoeba* sp. cells following cultivation with *B. pseudomallei* biofilm is the consumption of non-encapsulated biofilm cells of *B. pseudomallei* by the amoebae after the dispersal of the biofilm. The presence of amoebae is crucial for maintaining nutrient cycling and balancing bacterial populations in ecosystems^30^. *Acanthamoeba* sp. may feed on extracellular polymeric substances (EPSs) in *B. pseudomallei* biofilm structure including capsular polysaccharides (CPS), exopolysaccharide, proteins, or lipids^31^.

The levels of adhesion of non-encapsulated biofilm cells of *B. pseudomallei* H777, M10 and C17 to *Acanthamoeba* sp. is consistent with that of our previous study which has suggested the biofilm phenotypes of *B. pseudomallei* on initial adhesion and invasion in human lung epithelial cells^18^. As environmental predators, trophozoites of *Acanthamoeba* spp. approach different microbes using their universal receptors to bind with various bacterial surface components including capsules, peptidoglycan, lipopolysaccharide and β-(1–4)-N-acetylmuramic acid^20^. The actively grazing of *Acanthamoeba* sp*.* towards and target *B. pseudomallei* cells provide additional evidence for the amoebae-bacteria interactions. While, a mass spectrophotometry-based metabolomics approach demonstrated that *Burkholderia agricolaris* and *B. hayleyella* use chemotaxis to actively search for their host, the social amoeba, *Dictyostelium discoideum*^32^*.* However, further work should be undertaken to investigate how *Acanthamoeba* sp*.* attack *B. pseudomallei* to widen the understanding of the ecological interaction that may transform an environmental saprophyte to a potential pathogen.

Bacteria subject to attack by protozoa have evolved defensive mechanisms that allow them to survive within protozoa. These mechanisms also pre-adapt them as opportunistic pathogens to escape the harmful attentions of phagocytes^33–35^. Ours is the first study using UHPLC ESI-QTOF-MS/MS-based metabolic profiling to investigate the differences between *B. pseudomallei* cells following interactions with amoebae and cells grown in LB without the presence of amoebae. A remarkably elevated amount of 8-*O*-4´-diferulic acid was detected in *B. pseudomallei* after repeated encounters with amoeba. Nevertheless, colonies of *B. pseudomallei* H777 after three passages in *Acanthamoeba* sp. demonstrated similar morphology.

Amoebae and mammalian phagocytes share core mechanisms and molecular processes concerning phagocytosis and intracellular killing of pathogens^33^. Bacteria that can evade the digestion process to survive in amoebae may use similar mechanisms to avoid or survive in nonphagocytic and mammalian phagocytic cells. Therefore, *Acanthamoeba* is recognized for its influence on the evolution, persistence, and transmission of potential human pathogens^20,36^. Bacterial pathogens engage antioxidant strategies using superoxide dismutase and catalase to neutralise reactive oxygen species such as superoxide (O_2_^−^), hydrogen peroxide (H_2_O_2_) and hydroxyl (HO·), which are crucial pathogen-eradication mediators^24^. *Burkholderia pseudomallei* exhibits superoxide dismutase activity to detoxified the superoxide for its intracellular survival and virulence^37,38^. Likewise, ferulic acid and dimers of ferulic acid, commonly obtained from plants, have antioxidant properties as superoxide-scavenging molecules^39–41^. The detection of 8-*O*-4′-diferulic acid, a superoxide scavenging metabolite, in *B. pseudomallei* passaged in amoebae may offer preliminary insights into a possible strategy for evading amoebae-mediated destruction and enhancing survival within host cells. This result corroborates the findings of much of the previous work by Wan et al.^42^ that demonstrated the antioxidant defence in *V. cholerae* by utilizing catalase to scavenge reactive oxygen species*.* Furthermore, *V. cholerae* biofilms produce pyomelanin pigment and reactive oxygen species correlated with resistance against *A. castellanii* predation^43^. In addition, as well as replicating in the amoeba *A. castellanii*, intracellular *V. cholerae* could ultimately return to the aquatic habitat using quorum sensing involving a *Vibrio* polysaccharide^44^. *Vibrio cholerae* that survived intracellular killing might gain specific strategies that enhance their hypervirulent performance in human hosts. Hence, environmental *V. cholerae* that have passed through protozoa may be preadapted to become human pathogens^23^. Furthermore, extensive investigations are required to fully comprehend the role of the metabolic changes observed in *B. pseudomallei* following passage through amoebae in enhancing *B. pseudomallei* survival.

A possible limitation of our metabolomics study is that we only used short periods of 4.5 h for the thrice-passaged *B. pseudomallei* in *Acanthamoeba* sp. to obtain the intracellular-surviving bacteria. In addition, culture of the liberated internalised bacteria to obtain sufficient bacterial cells for the MOI 100 co-cultivation and metabolomics analysis might have influenced the results. Metabolites in *B. pseudomallei* may be altered during different stages of bacterial growth^45–47^ leading to the appearance of similar metabolites between treated and control groups. Further work is required to investigate metabolites produced by amoeba-internalised bacteria without their subsequent growth in bacterial culture medium^48^. Moreover, in the biofilm grazing observations, conducting an enumeration of *B. pseudomallei* H777, M10 and C17 both with and without co-cultivation with *Acanthamoeba* sp. may offer valuable insights into the impact of amoeba grazing.

To the best of our knowledge, this is the first report on interactions between *B. pseudomallei* biofilm and *Acanthamoeba* sp. The principal theoretical implication of this study is that *B. pseudomallei* biofilm provides general protection against grazing by *Acanthamoeba* sp. Metabolomic analysis identified 8-*O*-4′-diferulic acid, a superoxide scavenging metabolite, that may play a role in predator-driven *B. pseudomallei* adaptation. The ability of *B. pseudomallei* to resist digestion by free-living amoebae may preadapt the bacterial pathogen to life as an intracellular pathogen.

## Materials and methods

### Ethics statement

*Burkholderia pseudomallei* H777 (from Melioidosis Research Center, Khon Kaen University) had been collected as a part of the study of the epidemiology of *B. pseudomallei* approved by the Khon Kaen University Ethics Committee for Human Research (HE490324). Patient cannot be identified as the isolates de-identified when we received them. All procedures were conducted following the appropriate guidelines and regulations.

### Bacterial strains

*Burkholderia pseudomallei* H777, M10 and C17 isolates^18,49^ (Table 1) from glycerol stock at -80 ºC were cultured on Ashdown’s agar at 37 °C for 48 h. A single colony was cultured in 5 mL Luria Bertani (LB) broth at 37 °C, 200 rpm for 16–18 h before dilution to an optical density (OD_600_) 0.1 (≈ 1 × 10^7^ CFU/mL) for 2% inoculum in fresh LB broth for 8 h to reach log phase. Subsequently, the bacterial cells were harvested and washed twice with sterile Page’s modified-Neff’s amoeba saline (PAS)^50^ at 3000 × g for 5 min and adjusted to OD_600_ = 0.1 for the co-cultivation experiment with amoebae. *Burkholderia pseudomallei* in LB at OD_600_ = 0.8–0.9 was used as the starter inoculum for biofilm establishment^18,51^.

*Escherichia coli* grown in LB broth at 37 °C, 200 rpm for 16–18 h were harvested and washed twice with PAS and were used to feed *Acanthamoeba* sp. to maintain the trophozoite stage^27^.

### Cultivation of amoebae

*Acanthamoeba* sp. previously isolated from a *B. pseudomallei-*positive soil sample in Khon Kaen, Thailand^27^ was used in this study. The amoebae from soil stock were cultured on a non-nutrient agar plate with the addition of 0.03% trypticase soy broth (TSB) and *E. coli* as food*.* Plates were observed daily under a stereo microscope until the amoebae cells reached 70% confluence. The cells were then harvested and washed with PAS for further investigation.

*Acanthamoeba* sp. cells were grown in a gradually increased kanamycin concentration (from 30 to 300 µg/mL) administered via daily changes of PAS for 10 days^27^ to induce tolerance to 300 µg/mL kanamycin. The kanamycin pre-treated amoebae were used in co-cultivation experiments.

### Monitoring adhesion and intracellular survival of two *B. pseudomallei* biofilm phenotypes

To monitor adhesion, the first step in the process of bacterial internalization of *B. pseudomallei* biofilm phenotype, amoeba cell suspensions in PAS (1 × 10^3^ cells/well) were seeded in a 24-well tissue-culture plate and allowed to attach to the bottom of the culture plate for 15 min. Co-culture of non-encapsulated biofilm cells of *B. pseudomallei* H777 (wild-type strain) or M10 (biofilm-defect strain) with *Acanthamoeba* sp. was performed by adding mid-log suspensions of *B. pseudomallei* at a multiplicity of infection (MOI) of 100 and incubation for 1 h at 30 °C. Non-adherent bacteria were then removed by five gentle washes using PAS. Subsequently, amoeba cells were then lysed with 0.1% (v/v) Triton X-100 in PBS pH 7.4 for 20 s to liberate adherent bacteria. The percentage of adhered bacteria was calculated from the number of colony-forming units (CFUs) after incubated for 48 h at 37 °C on Ashdown’s agar using a drop plate technique, compared to the number of CFUs of the inoculum.

Microscopic observations of the adhesion experiments were also performed using a sterile coverslip placed in a 24-well plate before *Acanthamoeba* sp. and *B. pseudomallei* were co-cultured for 1 h. After washing with PAS, both amoeba and bacterial cells adhering to the coverslip were fixed with 1.25% (v/v) glutaraldehyde (EM grade; Electron Microscopy Sciences, Hatfield, PA) and stained with 0.1% crystal violet for 3 min. After washing with PBS buffer and air-drying at room temperature for 60 min, the coverslip was then mounted onto a glass slide and examined under a bright field microscope (Nikon, Eclipse Ni, Japan) at 100 × oil-immersion objective magnification.

To examine the intracellular survival of *B. pseudomallei*, bacteria were co-cultivated with *Acanthamoeba* sp. at MOI 100 for 1 h. After the non-adherent bacteria were removed, the amoebae were washed 3 times with PAS followed by the kanamycin protection assay to eradicate the extracellular bacteria using kanamycin at 300 µg/mL for 30 min. Subsequently, the initial internalized bacteria at 1.5 h post-infection (p.i.) and the intracellular survival after 3 h further incubation (4.5 h p.i.) were liberated using Triton X-100 and counted. The percentage of the internalized *B. pseudomallei* were reported compared to the inoculum.

To investigate the interaction between *B. pseudomallei* and *Acanthamoeba* sp., time-lapse video recording was performed. The amoeba cells were co-cultured with mid log-phase *B. pseudomallei* at MOI of 100 for 1 h at 30 °C. The interactions were then observed under an inverted-light microscope for another 10 min, 600 × magnification. The time-lapse video was displayed at 32 × speed.

### *Burkholderia pseudomallei* biofilm formation versus *Acanthamoeba* sp. grazing

The liquid–air interface of *B. pseudomallei* biofilm was established using a 1 mL inoculum on a sterile glass coverslip in a 24-well plate with an Amsterdam Active Attachment (AAA) model at 37 °C for 24 h and 48 h^51^. The 24-h and 48-h pre-formed biofilms on the glass lid were washed once with sterile PBS before approximately 1 × 10^3^ *Acanthamoeba* sp. cells/well in PAS were inoculated and incubated at 30 °C for an additional 24 h. The 48-h and 72-h biofilms challenged with the amoeba and controls on the glass coverslip were stained with 50 µg/mL FITC-ConA (Sigma, Saint Louis, Missouri, USA) for 20 min, fixed with 2.5% glutaraldehyde in PBS at room temperature for 3 h, washed three times with PBS and mounted with 80% glycerol. The biofilm structure was examined under confocal laser scanning microscope (CLSM, LSM 800, Carl Zeiss, Jena, Germany). The biofilm intensity and biomass of adherent cells were analysed using ZEN (version 2.1 blue edition) and COMSTAT software (version 2.1).

To assess the quantity of amoeba cells after their co-cultivation with pre-formed *B. pseudomallei* biofilms, the suspension from each well was collected and the number of amoeba cells was counted using a haemocytometer.

### Sample preparation for LC–MS metabolite profiling

In our previous study, we observed that *B. pseudomallei* survived for up to 3 h post-infection but complete eradicated by 6 h within *Acanthamoeba* sp.^27^. We hypothesized that during this time frame, internalized *B. pseudomallei* might express certain metabolites crucial for its survival. The internalised *B. pseudomallei* H777 in *Acanthamoeba* sp. at 4.5 h p.i. were therefore liberated and grown on Ashdown’s agar at 37 °C for 48 h. A single colony was then taken and grown in 10 mL LB broth at 37 °C, 200 rpm for 6 h to achieve log-phase growth. The bacteria were again co-cultured with amoebae at MOI 100 for two additional cycles. After the third co-cultivation, the internalised bacterial cells were liberated, grown on Ashdown’s agar, and recovered in 10 mL LB broth and harvested for metabolomic analysis. Cells were washed three times with cold PBS pH 7.4 and centrifuged at 3,000 × g for 5 min at 4 °C before cell density was adjusted to OD_600_ 0.5–0.6 (≈ 1 × 10^8^ CFU/mL). The bacterial suspension was then centrifuged at 10,000 × g at 4 °C, for 10 min to obtain bacterial pellets. In parallel, *B. pseudomallei* H777 was grown on Ashdown’s agar and in LB broth for 3 rounds before harvested by centrifugation as an untreated control. The bacterial pellets were kept at − 80 °C for further metabolite extraction.

### Metabolite extraction

The frozen *B. pseudomallei* cell pellets were resuspended in 200 µL of ice-cold methanol: water (1:1, v/v) and transferred to cryotubes containing 0.3 g of 0.5 mm sterile glass beads. Aqueous metabolite extraction was performed using a bead beater (OMNI bead rupture 24, Georgia) at 4.5 m/s for 30 s at 25 °C for 2 cycles followed by centrifugation at 20,000 × g, 4 °C for 10 min. The supernatant was transferred into a new tube before mixed with 200 of ice-cold chloroform, incubated on ice with interval vortex mixing every 3 min. After 20 min, the mixture was centrifuged at 20,000 × g, 4 °C for 10 min. The aqueous phase was dried using a CentriVap concentrator (LABCONCO, Missouri) at 45 °C for 3–4 h. Each sample was stored at -80 °C for further UHPLC-ESI-QTOF-MS/MS analysis. The dried extracts were reconstituted with 100 µL solvent mixture of water: acetonitrile (1:1, v/v), sonicated at room temperature for 10 min for 2 times, and centrifuged twice for 15 min at 20,000 × g, 4 °C^47,52^. From each sample, 15 µL was collected, pooled, and then used as a quality control (QC) sample.

### LC–MS data acquisition

Metabolite profiling of *B. pseudomallei* samples during interaction with *Acanthamoeba* sp. was carried out using ultra-high-performance liquid chromatography coupled with electrospray ionization (ESI)-quadruple time-of-flight mass spectrometry (UHPLC ESI-QTOF-MS/MS) (Bruker, Germany) at Khon Kaen University Phenome Centre (KKUPC). In brief, the aqueous phase extracts of samples were analysed on a reverse-phase liquid chromatography platform using a Bruker intensity HPLC C18 (2.1 × 100 mm, 2 µm column). The column temperature was set at 40 ºC and the autosampler temperature was set at 4 °C. Mobile phase A was 100% water with 0.1% formic acid (FA) and mobile phase B was 100% acetonitrile with 0.1% FA. The flow rate was set at 0.35 mL/min and the elution gradient was set as follows: 99% A (0.0–2.0 min, 0.25 mL/min), 1% A (2.0–20.0 min, 0.25 mL/min), 99% A (20.1–28.3 min, 0.35 mL/min), 99% A (28.5–30.0 min, 0.25 mL/min). Two µL of samples were injected for both positive and negative ionisation polarity mode. The MS temperature was set at 220 °C, desolvation gas 8 L/min. Sodium formate solution (2 mM sodium hydroxide, 0.1% FA and 50% isopropanol) was directly injected as an external calibrant with the flow rate of 0.5 µL/min. The capillary voltage in positive and negative ionization polarity modes were 4000 and 4500 V, respectively. The data scan was set to mass range 50–1500 m/z)^53^.

### Data pre-processing, metabolite assignment and multivariate statistical analysis

Raw data were imported to MetaboScape 7.0.1 software (Bruker, Massachusetts, US) for data pre-processing. In MetaboScape, the bucket table parameters were generated by using T-ReX _3D (LC-QTOF) workflow. Detection of molecular features was set 1500 counts of intensity threshold with a minimum peak length of 8 spectra. Assignment of metabolites was performed by comparing the MS/MS fragmentation patterns of detected features against the public database, human metabolome database (HMDB), METLIN, Bruker Metabobase and LipidBlast database. The level of assignment (LoA) included (1) accurate mass matched to database indicating tentative assignment, (2) accurate mass matched to database and tandem MS spectrum matched to in silico fragmentation pattern, (3) tandem MS spectrum matched to database or literature, (4) retention time and the molecular mass matched to standard compound, and (5) MS/MS spectrum matched to standard compound. The multivariate statistical analysis, including principal component analysis (PCA) and orthogonal signal correction-projection to latent structures-discriminant analysis (O-PLS-DA), were conducted using the Pareto scaling method in SIMCA software version 14.1 (Umetrics, Umeå, SE).

### Univariate statistical analysis

Statistical analysis was performed in IBM SPSS statistics for Windows version 28 (SPSS Inc., Chicago, USA). Data was analysed from three independent experiments. The data was illustrated as a graph of the mean ± standard deviation (SD), using Graph Pad prism 5 (GraphPad Software Inc., California, USA). One-way ANOVA followed by Tukey post hoc test were used to identify significant differences among groups. Comparisons of metabolite spectra intensities were performed using a non-parametric test, the Mann–Whitney U test. A statistically significant difference was considered at *p* < 0.05.

### Supplementary Information

Supplementary Figures.

Supplementary Video 1.

## Data Availability

The datasets used and/or analysed during the current study available from the corresponding author on reasonable request.
