# Supplementary material for: Burkholderia pseudomallei biofilm resists Acanthamoeba sp. grazing and produces 8-O-4′-diferulic acid, a superoxide scavenging metabolite after passage through the amoeba
Source: Sci Rep. 2023 Oct 3;13:16578. doi: 10.1038/s41598-023-43824-1 (PMC10547685; doi:10.1038/s41598-023-43824-1)
Supplement: Supplementary file 1 — Supplementary Figures. [file 41598_2023_43824_MOESM1_ESM.docx]

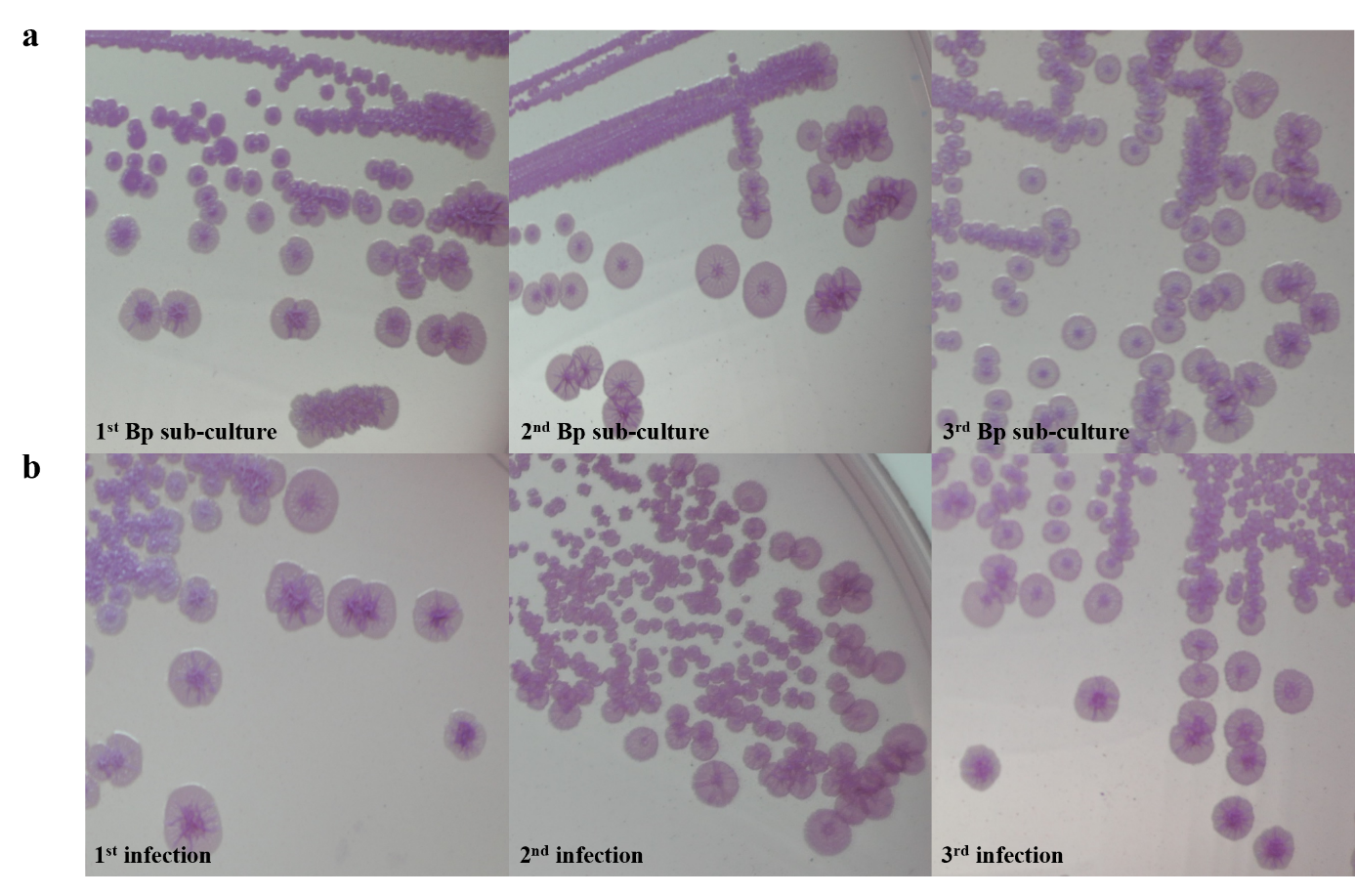


**Supplement Figure 1. The colony morphology of *B. pseudomallei* H777 was observed after it was passaged through *Acanthamoeba* sp. three times on Ashdown's agar.**

The colony morphology of control *B. pseudomallei* H777 after being sub-cultured in LB broth (**a**) and after each of the three passages through *Acanthamoeba* sp. (**b**) were comparable**.**


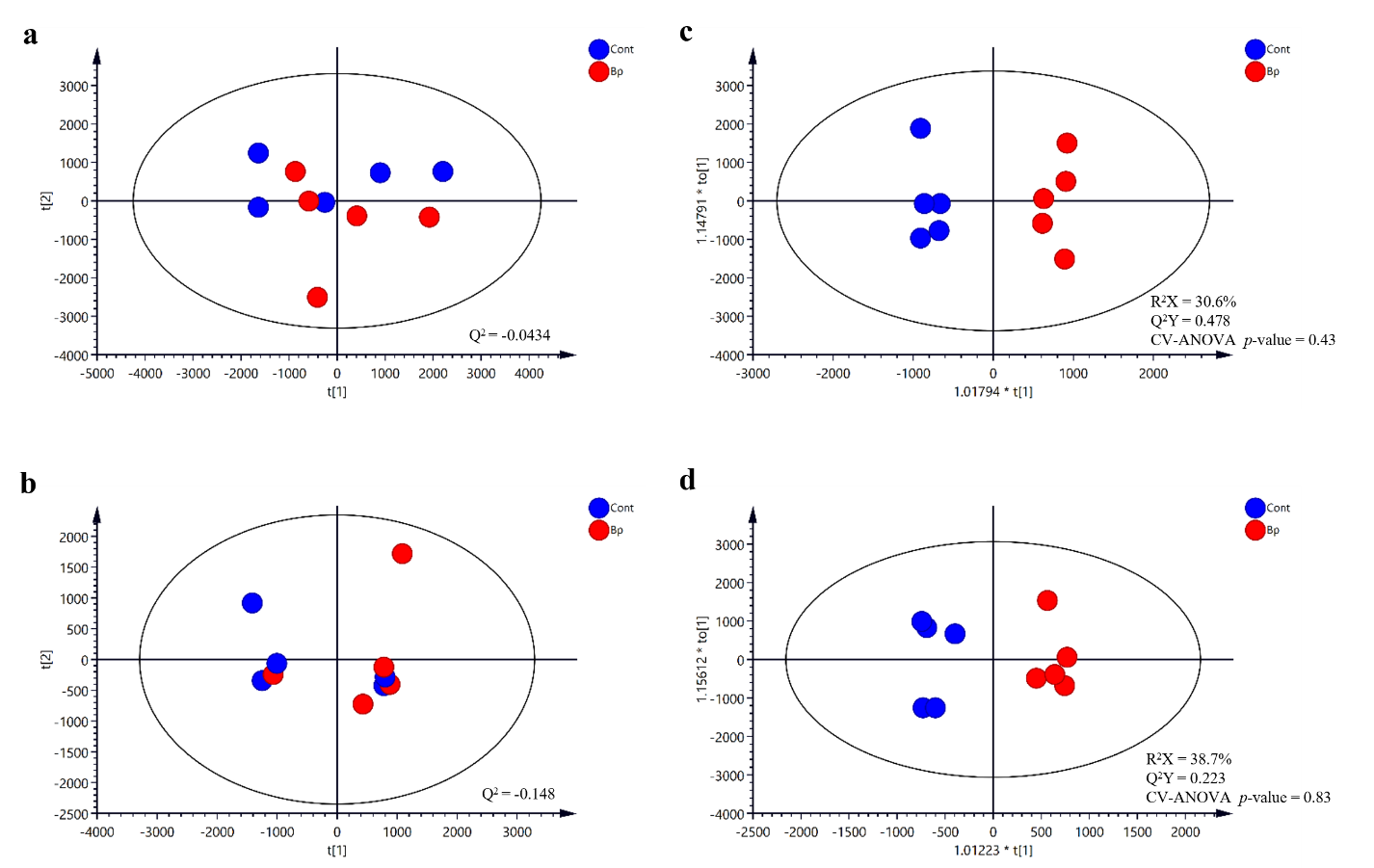


**Supplement Figure 2.** **PCA and O-PLS-DA plot cross-validated score plots between control (*B. pseudomallei* H777 without *Acanthamoeba* sp.) and experimental groups (*B. pseudomallei* H777 co-cultured with *Acanthamoeba* sp.)**

PCA plots of positive (**a**) and negative (**b**) ionisation mode. O-PLS-DA plots of positive (**c**) and negative (**d**) ionisation mode. The data were Pareto scaled.
