# Supplementary material for: Burkholderia pseudomallei biofilm resists Acanthamoeba sp. grazing and produces 8-O-4′-diferulic acid, a superoxide scavenging metabolite after passage through the amoeba
Source: Sci Rep. 2023 Oct 3;13:16578. doi: 10.1038/s41598-023-43824-1 (PMC10547685; doi:10.1038/s41598-023-43824-1)
Supplement: Supplementary file 2 — Supplementary Video 1. [file 41598_2023_43824_MOESM2_ESM.pptx]

## Slide 1
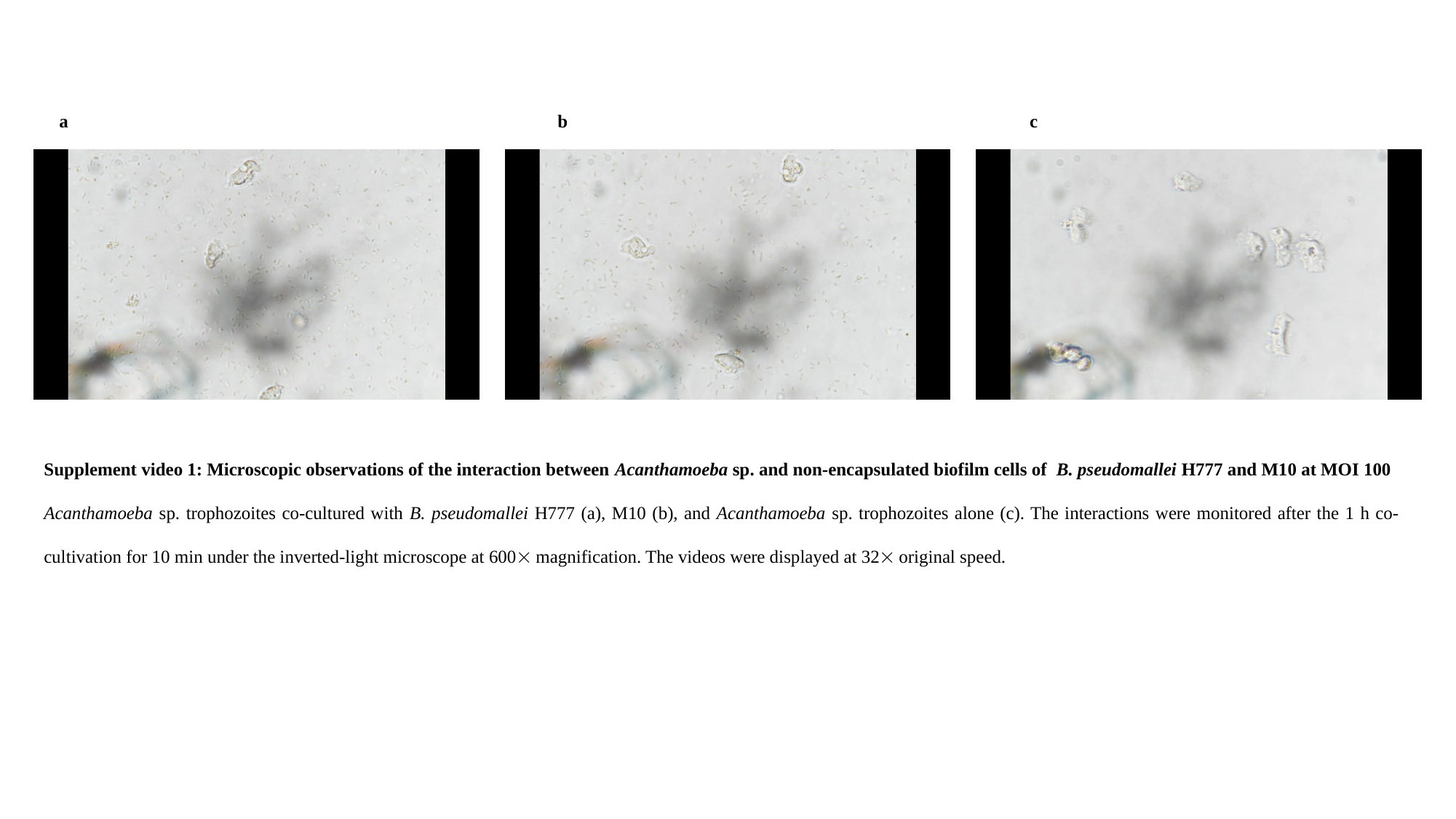

c
a
b
Supplement video 1: Microscopic observations of the interaction between Acanthamoeba sp. and non-encapsulated biofilm cells of B. pseudomallei H777 and M10 at MOI 100
Acanthamoeba sp. trophozoites co-cultured with B. pseudomallei H777 (a), M10 (b), and Acanthamoeba sp. trophozoites alone (c). The interactions were monitored after the 1 h co-cultivation for 10 min under the inverted-light microscope at 600× magnification. The videos were displayed at 32× original speed.
